# A 1.5-year randomized controlled trial comparing standard-sized implants and two diameters of mini-implants immediately loaded by mandibular overdenture: radiographic outcomes, short-term survival, and success rate

**DOI:** 10.1038/s41405-025-00369-x

**Published:** 2026-02-04

**Authors:** Heba M. Moftah, Mohannad H. Al-Saadi, Khaldoun Darwich

**Affiliations:** 1 Department of Removable Prosthodontics, Faculty of Dental Medicine, Damascus University, Damascus, Syria; 2 Department of Oral and Maxillofacial Surgery, Faculty of Dental Medicine, Damascus University, Damascus, Syria

**Keywords:** Gerodontics, Removable prosthodontics, Dental implants

## Abstract

**Objective:**

This study aims to evaluate the marginal bone level changes (MBLC), survival, and success rate of 4 mini-implants (MIs) of two different diameters versus two standard-sized implants (SIs) that were loaded functionally and immediately by a complete mandibular denture.

**Materials and Methods:**

The study comprised 29 participants with conventional complete dentures; they were randomly allocated into three groups. Group S included 11 participants who received two SIs of 3.75 mm diameter, group M3 included 8 participants who received four MIs of 3 mm diameter, and group M2.5 included 10 participants who received four MIs of 2.5 mm diameter. MBLC was evaluated on each implant’s mesial and distal sides after 18 months of loading. A paired t-test and one-way ANOVA were used for intragroup and intergroup comparisons, respectively (*P* ≤ 0.05). The evaluation of survival and success rates adhered to the criteria established by the International Congress of Oral Implantologists in Pisa (2007).

**Results:**

Immediate loading was employed in all cases. No failure occurred in any case. The success rate after 18 months was 90.91% in the S group, 90.63% in the M3 group, and 90% in the M2.5 group. MBLC after 18 months of loading were 0.76 ± 0.62 mm mesially and 0.63 ± 0.90 mm distally in the S group, 1.04 ± 0.72 mm mesially and 1.05 ± 0.84 mm distally in the M3 group, and 1.37 ± 0.73 mm mesially and 1.11 ± 0.68 mm distally in the M2.5 group.

**Conclusion:**

The SIs group showed better radiographic results and success rates than the MIs groups. As the diameter of the implant decreases, the mean MBLC increases. However, the results of the MIs groups were within acceptable limits. The survival rate was 100% in the three groups.

## Introduction

For edentulous patients, implant-retained overdentures may generally be a preferred option that promises to solve many of the limitations of conventional dentures [1] and have some benefits, such as the possibility of reduced alveolar bone resorption [1–4], as marginal bone level changes (MBLC) around implants is four times less than physiological bone resorption in the anterior mandibular region [5, 6]. Implants beneath mandibular overdentures result in better retention, improved masticatory efficiency, less bone resorption, and less muscle atrophy than conventional dentures [7, 8]. It is important to note that implants partially compensate for the loss of sensory function through osseoperception. The interocclusal tactility of bone-integrated implants is less than that of natural teeth. Nevertheless, this is still better than not receiving any treatment with implants [9].

Standard-sized implants (SIs) may not always be clinically feasible for anatomical reasons, the quantity and quality of the residual bone [10–13]. In some patients, treatment may require bone augmentation or expansion procedures [13, 14], which are invasive procedures that increase cost, time, and the risk of side effects [14]. Additionally, these procedures may increase morbidity and the likelihood of complications [15]. Additionally, some patients may refuse further surgical interventions or may not be suitable candidates for such alternatives [13]. As a result, small-diameter implants were developed. The first group of the International Team for Implantology (ITI) consensus in 2018 stated that small implants with a diameter of 3.5 mm or less can effectively support complete mandibular overdentures [16].

Immediate loading is becoming more popular among clinicians and patients in appropriate clinical situations. Patients desire a fixed prosthesis that replaces their missing teeth quickly and painlessly. Meanwhile, clinicians aim to reconstruct the patient’s dentofacial system using a fast, stable, and aesthetic approach [17]. For some patients, particularly those with complete edentulism, lengthy healing times can be intolerable from aesthetic, functional, psychological, and social perspectives [18, 19].

Many articles compare mini-implants (MIs) and two SIs but often focus on specific diameters of MIs [20] or vary diameters based on alveolar ridge width [21, 22]. Additionally, some studies compare MIs with immediate loading and SIs with conventional loading or different surgical techniques [6, 23], which can be problematic since flapless surgery isn’t always applicable. Many existing studies are retrospective [14], indicating a need for prospective, randomized studies. This study aims to evaluate the impact of implant diameter (3.75, 3, and 2.5 mm) on bone resorption, survival rates, and success rates of implants. The null hypothesis is no significant difference in MBLC, survival, or success rates between mandibular overdentures retained by two SIs versus four MIs of different diameters.

This study reports a significant gap in existing literature and, to our knowledge, is the first randomized controlled trial that compares various diameters of MIs to SIs while employing the same surgical and prosthetic protocol.

## Materials and methods

This parallel randomized controlled trial was approved by the Scientific Ethics Committee and the Council of the Faculty of Dental Medicine (No. 798, dated 28/8/2018), as well as by the Council of Scientific Research and Postgraduate Studies at Damascus University (No. 3394, dated 3/9/2018). All methods were performed in accordance with the Declaration of Helsinki. The study protocol was registered in the ClinicalTrials.gov database before starting the trial, under the number NCT03866031 (07/03/2019). The article was written according to the 2010 CONSORT standards for reporting randomized clinical trials [24].

The G-Power _3.1_ indicated that a minimum sample size of 7 participants in each group was needed for 80% power and a significance level of 0.05, which is widely recognized as acceptable power for research [25]. Randomization was conducted using a computer-generated sequence (via www.random.org), and participants were assigned to groups using sealed opaque envelopes. The same researcher, H.M., carried out all the stages related to randomization. The sample was collected from patients at the Faculty of Dental Medicine, Damascus University, between 2019 and 2022, who experienced problems with the retention of mandibular dentures and desired to receive implants.

The inclusion criteria were that the patient had a complete mandibular edentulism for at least six months before implantation, a class I Angle jaw relationship, the corresponding prosthesis was a complete denture, and sufficient bone for an implant of at least 10 mm in length and 3.75 mm in diameter. The exclusion criteria were any systemic disease that prevents surgery or affects the bones, such as patients who received radiotherapy or chemotherapy; patients with a disorder of calcium ion metabolism; uncontrolled diabetes; patients treated with steroids and immunosuppressive drugs; patients with osteoporosis; and those taking bisphosphonates intravenously for any period [26] or orally for more than 3 years [27]. Additionally, heavy smokers (more than 10 cigarettes per day), dysfunctional oral habits, psychological patients, and those with disorders of the temporomandibular joint.

Participants received a detailed information sheet regarding the study. After agreeing to participate, written informed consent was obtained from all participants. The study included 29 participants with conventional complete dentures. The mandibular dentures were converted to implant-retained overdentures following a functional-immediate loading protocol according to the three groups: group M2.5 and group M3, in which they were retained by four MIs of 2.5 mm or 3 mm in diameter, respectively (Inclusive, California, USA), self-tapping, one-piece, and ball attachments; and group S, in which they were retained by two SIs of 3.75 mm diameter (Intra-lock, Tapered implants, USA), self-tapping with two separate ball attachments.

The mandibular denture was examined to ensure it met academic standards for fitting, balanced occlusion, extension, maxillomandibular relationship, vertical dimension, and non-wearing anatomical teeth. If it did not, corrections were made or a new denture was created. A waiting period of at least two weeks was observed before implantation.

A mandibular denture replica with gutta-percha markers was used as a radiographic stent and converted into a CBCT-based surgical stent. Following Misch’s guidelines [28], two SIs were placed near the canines and four MIs in A-B-D-E between the two mental foramina. The guide was sterilized through autoclaving.

Each patient received 2 g of amoxiclav one hour before surgery. A one-minute oral rinse was performed using 0.12% chlorhexidine, and a povidone solution was applied for disinfection around the mouth. The participant was anesthetized with infiltration into the mental foramen and anterior lingual region with 2% lidocaine and 1:80,000 adrenaline. A full-thickness flap was achieved in all groups. Irregular alveolar bone was vertically trimmed to create a flat-like alveolar crest, especially in the MIs groups, where MIs needed to be at the same height. Unlike two-piece implants, MIs are one-piece, so the attachment neck length cannot be adjusted. The instructions and armamentarium from each manufacturer were followed to attain at least 30 Ncm torque, considering that the bone between the two mental foramina is often of high density. Parallelism was consistently verified using parallel pins, particularly in the MIs groups, as non-parallelism is less tolerated in a one-piece design [13]. The manufacturer’s manual states that the metal housing allows for up to 30 degrees of angular divergence between seated implants; however, this substantial divergence leads to considerable wear of the O-rings. Initially, the implants were mechanically inserted at 25 RPM, then manually inserted using a torque wrench to attain a torque of no less than 30 Ncm and no more than 45 Ncm for MIS and no more than 60 Ncm for SIs. The ball attachments are tightened to a torque of 25 Ncm within SIs immediately after the implant insertion and before suturing. All surgical procedures were performed by H.M. and M.A. Each patient received a single dose of diclofenac sodium (75 mg IV) and dexamethasone (8 mg IV) postoperatively. It was followed by a course of amoxiclav (1 g tap/12 h/7 d), diclofenac sodium (75 mg tap/12 h/7 d), bromelain (10,000 IU/8 h), and 0.12% chlorhexidine mouthwash (8 h/7 d).

All SIs and MIs were functionally immediately loaded by the mandibular denture within 48 hours of implantation using unsplinted O-ring abutments for simplicity and cost-effectiveness in all cases. The surgical incision was covered with a sterile rubber piece featuring holes at each O-ball head to prevent wound contamination with prosthetic materials. The position of the O-ball head was marked on the intaglio surface of the denture by lining it with a pressure indicator paste material. The denture was then relieved to create a suitable room for the O-ring housings. Metal housings were placed on each SI or MI, ensuring that blockout shims had fully covered the exposed neck of each implant beneath the O-ball head. The denture was placed in the patient’s mouth, and a passive fit over metal housings and handicap spots was checked by marking the roofs of the housings with a color transfer applicator. The denture was punched to let the excess acrylic resin get out. Finally, the O-ring housings are fixed into the denture using an autopolymerizing acrylic. The patient was instructed to close with normal pressure into a centric occlusion until the acrylic resin setting. The overdenture was then trimmed and polished. Finally, the patient was strictly instructed to keep the overdenture in place for the first 48 hours post-loading to prevent gingival overgrowth, eat soft food during the first month, and not eat without the overdenture. The surgical sutures were removed after 7-10 days. All prosthetic and follow-up procedures were performed by H.M. Blinding was impossible.

**Fig. 1:**
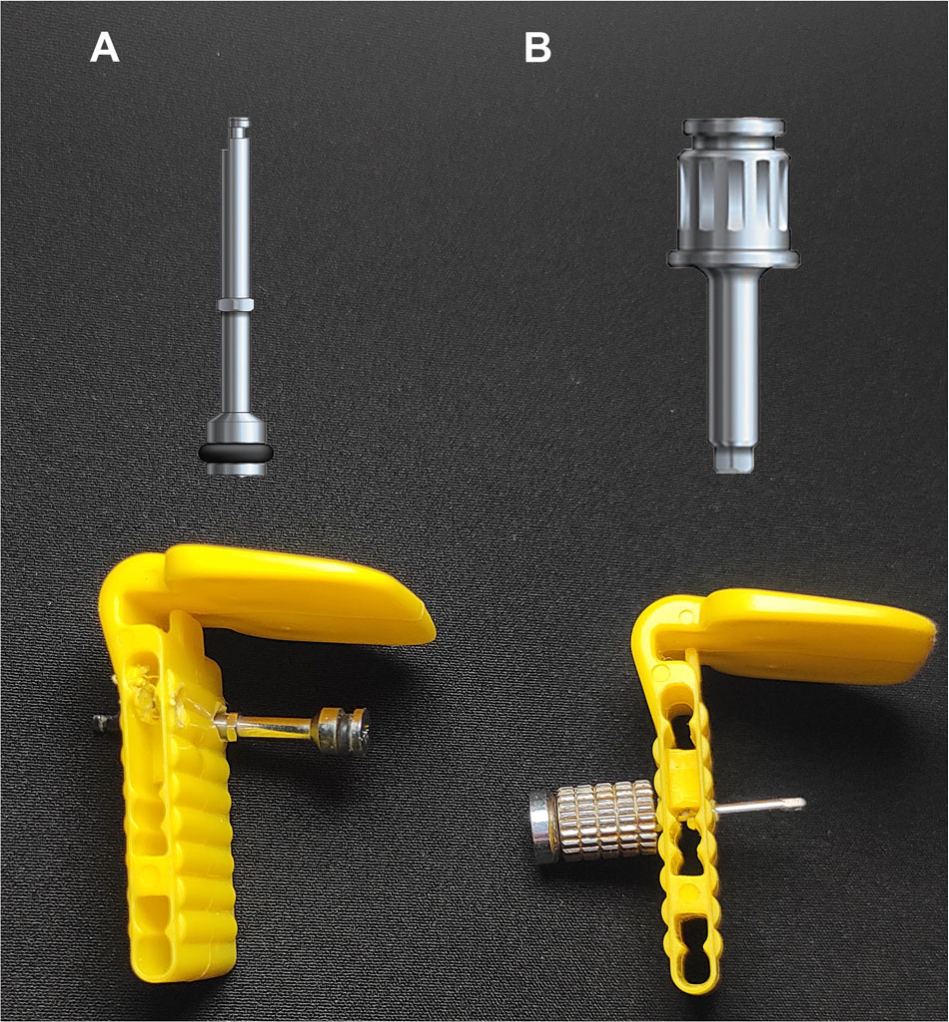
Modified X-ray sensor holder for parallel periapical images of mandibular implants. **A** for mini-implants, **B** for standard implants.

**Fig. 2:**
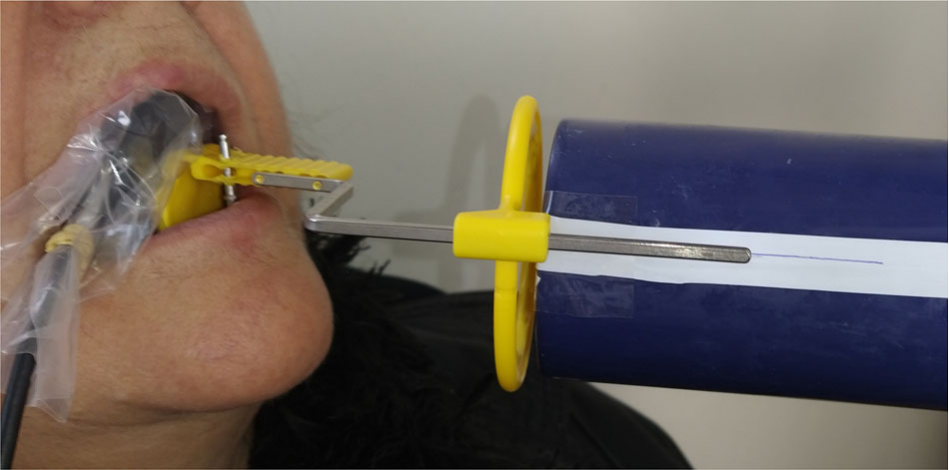
Parallel periapical X-ray image using the sensor holder. The X-ray beam was aligned perpendicular to the sensor using the metal arm and circular guide.

**Fig. 3:**
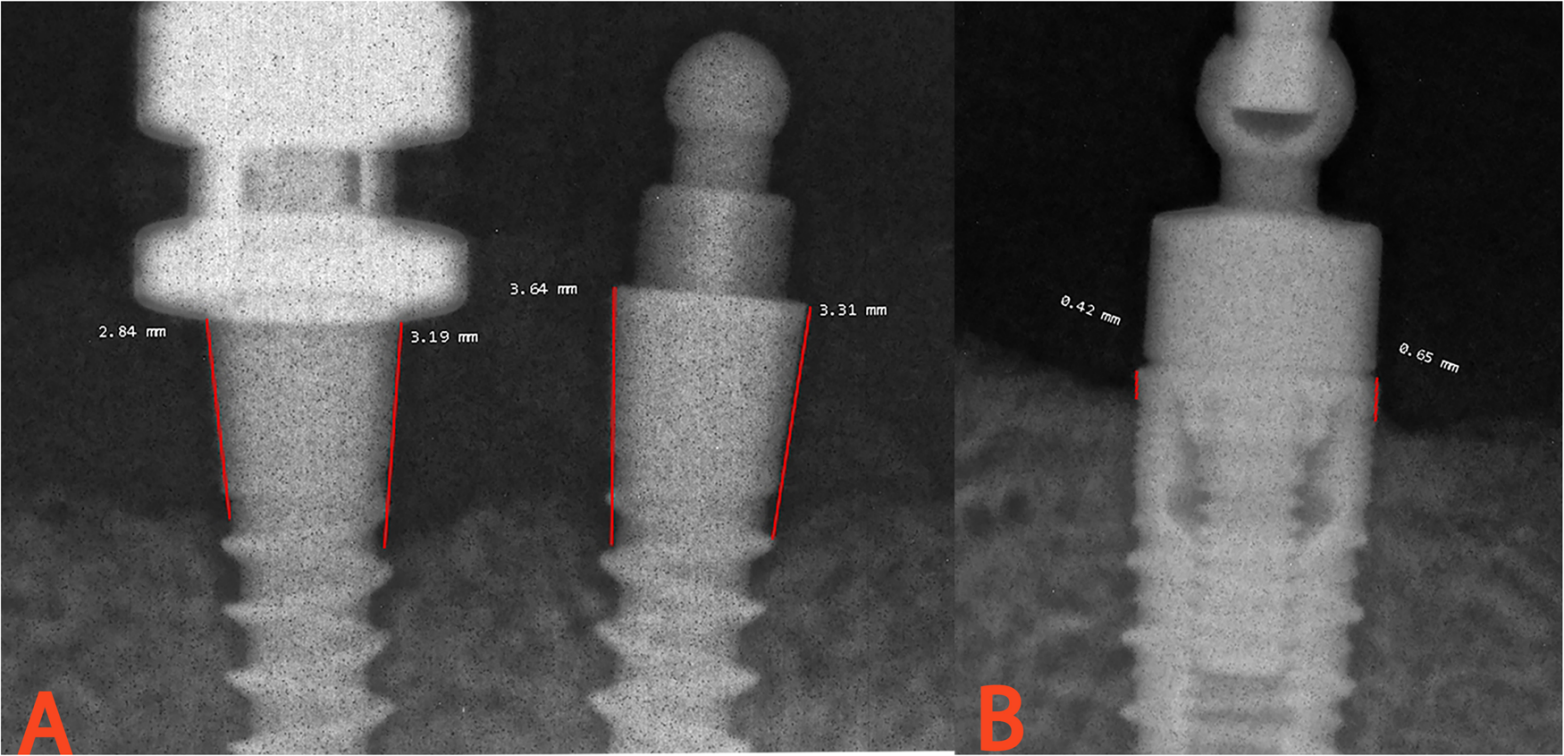
Marginal bone level measurement around mandibular implants. Distance between the highest point of implant-bone contact and: **A** the ball attachment shoulder of the mini-implant, **B** the shoulder of the standard implant.

Periapical images were taken at 1 week, 3, 6, 12, and 18 months after loading (T0, T3, T6, T12, and T18) using an X-ray device (France, De Gotzen, Xmind, intraoral X-ray) and an intraoral X-ray sensor (Italy, Trident, I-view). The X-ray holder for parallel images was modified to allow repeating images for each participant in the same position. It was accomplished by drilling two sensor holder pieces using the appropriate driver; one piece has a hole designed for the ball attachment driver used by the SIs group, while the other has a hole designed for the MIs driver (Fig. 1). A line was drawn in the middle of the X-ray cone to guide it parallel to the metal arm of the X-ray holder, ensuring that the beam was perpendicular to the X-ray sensor. To maintain a consistent distance between the X-ray nozzle and the X-ray sensor, the circular plastic piece was always placed in the middle of the metal arm (Fig. 2). The marginal bone level was measured using MicroDicom (Version 2022.3) software. The image was enlarged by 120% to ensure accurate distance measurement and was calibrated by a reference distance whose dimensions are pre-known for the actual measurement. Calibration was performed for each MI separately in the image with two MIs. The marginal bone level was determined by measuring the distance between a reference point (the shoulder of the SIs or the ball attachment shoulder of the MIs) and the highest point of implant-bone contact. Measurements were performed on the mesial and distal sides of each implant (Fig. 3). Marginal bone level at follow-up times (T3, T6, T12, T18) was calculated as the difference between the means and the baseline (T0).

The Consensus criteria established by the International Congress of Oral Implantologists (ICOI) in Pisa (2007) were followed to determine the survival and success rates. Success (optimum health) was defined as: (a) no pain or tenderness upon function, (b) zero mobility, (c) less than 2 mm radiographic bone loss from initial surgery, and (d) no exudates history. Satisfactory survival was defined as: (a) no pain on function, (b) zero mobility, (c) 2–4 mm radiographic bone loss, (d) no exudates history [29].

Statistical analyses were conducted using SPSS for Windows (Version 22.0, IBM Corp., Armonk, NY). Descriptive statistics were performed to obtain the mean and standard deviation of MBLC (mm) on mesial and distal sides. The Kolmogorov-Smirnov test verified that the data follow a normal distribution. The intergroup comparison was made using one-way ANOVA followed by Bonferroni post hoc analyses. The paired t-test was used for intragroup comparison between each two follow-up times. The significance level was set at *P* ≤ 0.05. Intra-examiner reliability was evaluated using the intraclass correlation coefficient (ICC, two-way mixed-effects model, absolute agreement, single measures: ICC(3,1)). Values were interpreted according to Koo & Li [30] as follows : Poor less than 0.5, Moderate between 0.5 and 0.75, Good between 0.75 and 0.9, and excellent greater than 0.90 [30].

## Results

The study sample consisted of 29 participants, aged 49 to 82 years, divided into three groups based on the diameters of the applied implants (Table 1).

**Table 1:** shows the distribution sample according to the participants’ gender, ages, number of implants, and implant lengths.

Groups	Participates number			Aged (years)		Implants	
	Male	Female	Total	Mean	Standard deviation	Number	Length (mm)
2 Standard-sized implants of diameter 3.75 mm	7	4	11	67.7	6.9	22	10 or 11.5
4 Mini-implants of diameter 3 mm	3	5	8	69.5	6.8	32	10 or 13
4 Mini-implants of diameter 2.5 mm	3	7	10	67.4	10.9	40	10 or 13
All sample	13	16	29	68.1	8.2	94	

**Fig. 4:**
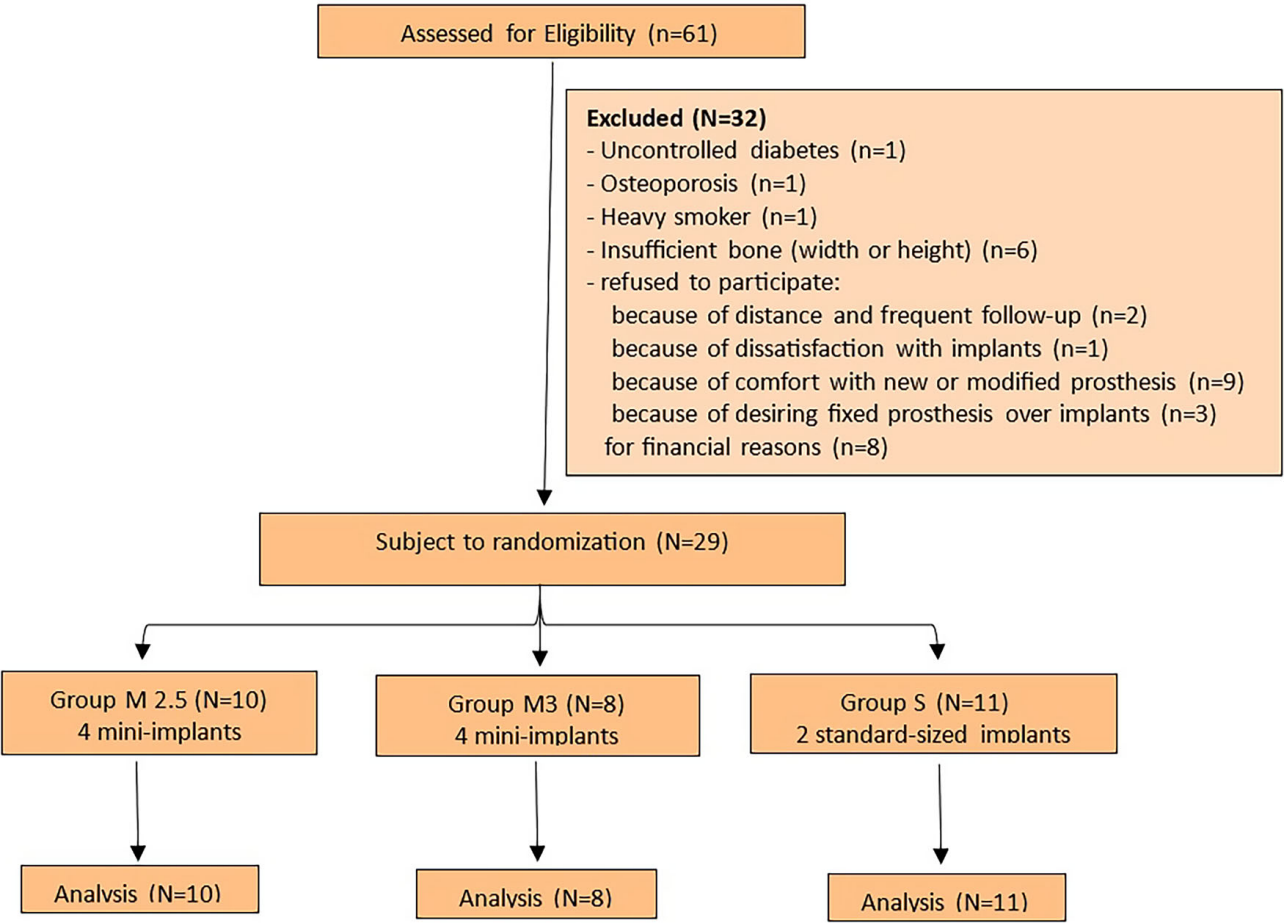
Flow diagram. Chart showing allocation of the enrolled participants and follow-up over the 18-month trial.

Functional immediate loading was successfully achieved in all samples and implants, except for one MI in one participant, where a torque of 30 Ncm was not achieved. This MI was only loaded non-functionally and immediately with soft acrylic for one month before the metal housing was fixed. It was included in the analysis, and given that it represents only one MI among the total sample, its impact on overall outcomes is expected to be minimal, consistent with the intention-to-treat principle. No participants dropped out, as shown in the flow chart (Fig. 4). The intra-examiner reliability for all radiographic measurements was excellent, with ICC values ranging from 0.9994 to 0.9999 (95% CI: 0.9938–0.9999) (Table 2).

**Table 2:** Intraclass correlation coefficients (ICC) for intra-examiner reliability of measurements.

Group	ICC (95% CI)	Interpretation*
S / Mesial	0.9995 (0.9949 - 0.9999)	Excellent
S/ Distal	0.9994 (0.9938 - 0.9999)	Excellent
M3 / Mesial	0.9999 (0.9997 - 0.9999)	Excellent
M3/ Distal	0.9997 (0.9987 - 0.9999)	Excellent
M2.5/ Mesial	0.9999 (0.99991 - 0.999994)	Excellent
M2.5/ Distal	0.9994 (0.9980 - 0.9999)	Excellent

*Values interpreted according to Koo & Li [30].

The intergroup comparison of the MBLC did not show a statistically significant difference, except between group S and group M2.5 on the mesial side at T18 follow-up, *p* = 0.005 (Tables 3, 4). An intragroup analysis of the three groups is provided in Table 5. The table indicates a statistically significant difference in all follow-up times for group M2.5 (*P* = 0.000). In group M3, no statistically significant differences were observed during the last period (T12-T18), with *P*-values of 0.668 and 0.154 for the mesial and distal sides, respectively. In group S, no statistically significant differences were observed on the mesial side during the last two periods (T6-T12 and T12-T18), with P-values of 0.885 and 0.883, respectively. No significant differences were observed between the two periods on the distal side (T3-T6 and T6-T12), with P-values of 0.639 and 0.301, respectively.

**Table 3:** Intergroup comparison of mean marginal bone resorption at the mesial and distal side using one-way ANOVA.

Follow-up times	marginal bone resorption on the mesial side (mm)									marginal bone resorption on the distal side (mm)								
	Group S		Group M3		Group M2.5		F-value	*P*-value	Significance differences	Group S		Group M3		Group M2.5		F-value	*P*-value	Significance differences
	Mean	Standard Deviation	Mean	Standard Deviation	Mean	Standard Deviation				Mean	Standard Deviation	Mean	Standard Deviation	Mean	Standard Deviation			
T3	0.49	0.50	0.18	0.23	0.29	0.34	2.806	0.104	-	0.39	0.52	0.30	0.39	0.19	0.24	2.114	0.127	-
T6	0.77	0.56	0.58	0.51	0.56	0.42	1.378	0.257	-	0.42	0.65	0.65	0.75	0.49	0.48	0.988	0.376	-
T12	0.77	0.67	1.02	0.73	0.89	0.54	0.996	0.373	-	0.50	0.83	0.95	0.89	0.84	0.59	2.357	0.100	-
T18	0.76	0.62	1.04	0.72	1.37	0.73	5.618	0.005	*	0.63	0.90	1.05	0.84	1.11	0.68	2.837	0.064	-

- **:** No statistically significant difference, *: Statistically significant difference *P* ≤ 0.05.

**Table 4:** Bonferroni Post-hoc analyses for marginal bone resorption on the mesial side at T18 follow-up.

Follow-up times	I	J	Difference between means (mm)	Standard error	*P*-value	Significance differences
T18	**Group S**	**Group M3**	-0.27	0.20	0.500	-
		**Group M2.5**	-0.61	0.19	0.005	*
	**Group M3**	**Group M2.5**	-0.34	0.17	0.148	-

- **:** No statistically significant difference, *: Statistically significant difference *P* ≤ 0.05.

**Table 5:** intragroup comparison of mean marginal bone resorption at the mesial and distal side using the paired t-test, *P* ≤ 0.05.

Groups	Comparison of means between two follow-up times	marginal bone resorption on the mesial side				marginal bone resorption on the distal side			
		Difference between means (mm)	*t*-value	*P*-value	Significance differences	Difference between means (mm)	*t*-value	*p*-value	Significance differences
**2 Standard-sized implants of diameter 3.75 mm**	T3–T0	0.49	4.556	0.000	*	0.39	3.532	0.002	*
	T6–T0	0.77	6.433	0.000	*	0.42	3.046	0.006	*
	T12–T0	0.77	5.446	0.000	*	0.50	2.847	0.010	*
	T18–T0	0.76	5.756	0.000	*	0.63	3.300	0.003	*
	T6–T3	0.28	3.541	0.002	*	0.03	0.476	0.639	-
	T12–T6	0.00	0.147	0.885	-	0.08	1.061	0.301	-
	T18–T12	−0.01	-0.149	0.883	-	0.13	3.445	0.002	*
**4 Mini-implants of diameter 3 mm**	T3–T0	0.18	4.425	0.000	*	0.30	4.357	0.000	*
	T6–T0	0.59	6.341	0.000	*	0.65	4.891	0.000	*
	T12–T0	1.03	7.781	0.000	*	0.95	6.023	0.000	*
	T18–T0	1.05	8.039	0.000	*	1.05	7.069	0.000	*
	T6–T3	0.42	5.432	0.000	*	0.35	3.239	0.003	*
	T12–T6	0.43	5.545	0.000	*	0.30	4.596	0.000	*
	T18–T12	0.03	0.433	0.668	-	0.10	1.460	0.154	-
**4 Mini-implants of diameter 2.5 mm**	T3–T0	0.29	5.342	0.000	*	0.19	5.014	0.000	*
	T6–T0	0.56	8.406	0.000	*	0.49	6.460	0.000	*
	T12–T0	0.89	10.499	0.000	*	0.84	9.025	0.000	*
	T18–T0	1.37	11.831	0.000	*	1.11	10.364	0.000	*
	T6–T3	0.27	5.019	0.000	*	0.29	5.128	0.000	*
	T12–T6	0.33	4.943	0.000	*	0.35	5.283	0.000	*
	T18–T12	0.48	5.718	0.000	*	0.27	4.452	0.000	*

- **:** No statistically significant difference, *: Statistically significant difference *P* ≤ 0.05.

After an 18-month follow-up period post-loading, the survival rate was 100% in the three groups. The success rates were 90.91%, 90.63%, and 90% in the S, M3, and M2.5 groups, respectively. No issues were reported regarding mobility, soft tissues, or pain and tenderness during function in any SIs or MIs. Only a few MBLC values exceeding 2 mm but not surpassing 3 mm were observed in the SIs group, and up to 3.43 mm in the MIs groups. These cases included two SIs in one participant from the S group, three MIs in two participants from the M3 group, and four MIs in two participants from the M2.5 group.

## Discussion

This randomized clinical trial evaluated survival, success rate, and MBLC in mandibular overdentures retained by SIs and MIs. These results showed no relationship between early failure and implant diameter, consistent with Olate et al. [31]. Overall survival was comparable between SIs and MIs over 18 months, aligning with Borges et al. and Sohrabi et al. (10, 36) and with reports of 100% survival in both SI and MI overdentures despite different surgery/loading protocols [23]. However, the results of the present study disagreed with lower survival reported by Šćepanović et al. (95.9% for four 1.8 mm diameter MIs) [32] and of Kämmerer et al. (96.25% for four MIs of 1.8 or 2.2 mm diameter) [33]. These disagreements appear largely attributable to the increased insertion torque, which likely led to fractures during insertion and bone necrosis (37, 38).

MBLC is a critical parameter of implant success [34]. Radiographic measurements at mesial and distal aspects are widely accepted for evaluating implant success [6, 23, 35, 36]. In addition to the ICOI criteria, acceptable bone resorption limits are typically ≤2 mm during the first year of loading, followed by ≤0.2 mm annually [29, 35–37]. In this study, mean MBLC did not exceed 1.5 mm after 1.5 years across groups, with broadly similar mesial/distal values. The only statistically significant between-group difference occurred at T18 on the mesial aspect for M2.5 versus S (*P* < 0.05), while the distal comparison approached significance (*P* = 0.064) (Tables 3, 4). Taking into consideration that MBLC observed in the MIs is partly due to a portion of the collar being embedded in the bone, this is implemented in some cases to align it with the level of other MIs, which helps prevent excessive bone trimming that can lead to increased resorption [38].

Several factors may have contributed to the high survival and success rates observed in this study. Possible explanations include: fabrication of a new maxillary and mandibular complete denture or modification of an existing one to meet academic standards concerning ridge extension, occlusion, fitting, and non-wear anatomical teeth, which achieves good tissue support and limits the intensity of transmitted forces to the implants, particularly when opposing complete dentures. Careful adherence to implantation and sterilization protocols. Achieving an insertion torque of at least 30 Ncm and no more than 45 Ncm in MIs and 60 Ncm in SIs to prevent MIs fracture or necrosis, as the anterior mandibular region has high-density bone. Appropriate design of the o-ring provides a self-cleaning effect, as well as the good design of the one-piece MIs, which does not contain a microscopic gap [38]. The O-ring in the three groups acts as a shock absorber, reducing loads and producing bending forces for shorter periods on implants [20], thus acting as a stress-breaker [39]. Selection of ball attachments with an appropriate gingival height to avoid inappropriate biomechanical loading and potential bone resorption [40, 41]. Adequate medication coverage before, during, and after surgery. Strict written and verbal instructions for implants and overdentures care. Regular monitoring and adjustment of occlusion and denture fit. While these factors are plausible contributors, they should be considered potential influences rather than confirmed causal determinants, as the study was not designed to isolate their individual effects.

Temporal patterns of MBLC differed by group. In group S, it was observed that most of the resorption occurred during T3 and stabilized between T6 and T18 on the mesial side. On the distal side, it stabilized between T3 and T12, and then a statistically significant increase in resorption was noted between T12 and T18. This could be attributed to a problem related to fit or occlusion caused by posterior bone resorption, which impacted the applied load on the implants, particularly on the distal side. On the mesial side, the mean resorption measured between T6 and T12 was 0.01 mm and showed an improvement of the same amount between T12 and T18; this can only be explained by bone formation. This finding agrees with many studies indicating that dental implants not only prevent alveolar bone resorption but also can promote bone formation [42, 43]. In the M3 group, MBLC continued and showed a statistically significant difference between each two follow-up times until T12, then it stabilized between T12 and T18. In contrast, the M2.5 group showed continued MBLC, with a statistically significant difference until T18 without any stabilization. This indicates that as the diameter decreases, the mean MBLC increases and shows a statistically significant difference across the follow-up periods. This is consistent with evidence that, while MI-retained overdentures are successful, SI-retained overdentures may yield more favorable radiographic outcomes [20].

Some authors have attributed the low bone resorption values to several factors, including the shock-absorbing O-rings, flapless surgery, which prevents bone resorption, and the self-tapping implant design, which increases the density of the bone in contact with the implants [21]. The self-tapping feature enhances primary stability based on the principle of osseous compression [44, 45]. In the present study, both implant systems featured the self-tapping design and O-rings. However, the implants were placed using flap surgery, which did not negatively impact bone resorption; instead, it facilitated the avoidance of a mental loop and better implant distribution. Achieving an insertion torque ≥30 Ncm also minimized micromotion, as movements >100 µm can induce bone resorption and fibrous tissue formation [46, 47]. Supporting this, a retrospective study reported that implants with low torque and delayed functional loading experienced greater bone loss than those placed with high torque and immediate functional loading [14].

Flap surgery was used in all groups to standardize the procedure, facilitate safe placement in knife-edged ridges, and improve visualization. Evidence indicates no significant differences in implant survival, postoperative pain, or morbidity between flap and flapless approaches [12, 48].

The literature lacks consensus on the link between keratinized gingiva and implant success [49]. Greenstein and Cavallaro noted that while keratinized tissue can be helpful, its necessity varies among patients, complicating predictions for tissue grafting [50]. In this study, it was aimed to preserve existing keratinized tissue, but refrained from additional procedures when little or none was present, as their benefits are unproven. This approach aligns with the main goal of MIs production to reduce the need for further surgeries, and our inclusion criteria were not limited to a specific type of mucosa, enhancing the generalizability of our findings.

There is no universal consensus on implant diameter classification [13, 51]. The Glossary of Oral and Maxillofacial Implants defines MIs as “implants fabricated of the same biocompatible materials as other implants but with smaller dimensions” [52]. Some studies consider MIs smaller than 3.3 mm [17, 34]. ITI consensus statements define implants ≤3.5 mm as narrow-diameter, with MIs being <3.0 mm (ITI 2014) or <2.5 mm (ITI 2018) [16, 53]. The classification scheme proposed by Al-Johany et al. and in a recent meta-analysis categorizes implants as extra-narrow (<3.0 mm), narrow (3.0– < 3.75 mm), standard (3.75– < 5.0 mm), and wide (≥5.0 mm) [51, 54]. In this study, both groups (M2.5 and M3) were initially classified as MIs for convenience; however, the M3 group aligns with narrow implants. The 3.75 mm diameter was chosen for the S group because it is recognized as Sis [16, 51, 53, 54]. This choice also enabled us to standardize the control group and include a larger number of suitable subjects than would have been possible with a larger diameter.

Study features: (1) The first comparative study between two diameters of MIs and SIs using the flap surgery and the immediate loading method, and it can be said that it is a comparison between mini-implants, narrow-diameter implants, and standard-sized implant implants. (2) The relatively good follow-up period is 1.5 years, as implant failure usually appears in less than a year. (3) Good follow-up by the participants and attendance at the tests without any dropouts, because the implantation procedure and participation were 100% of their desire.

Study limitations: (1) Radiography should ideally be performed right after implantation. However, the parallel method requires a film holder and X-ray sensor inside the mouth and some pressure on the floor of the mouth, which is challenging due to the participant’s pain and exhaustion after implantation or loading. As a result, the radiography was delayed until the pain lessened. (2) Using only one assessor to measure marginal bone level on digital X-rays instead of the preferred method with two calibrated assessors. But the assessor measured it twice at two different times to prevent error or personal bias, and in the event of a major difference in the evaluation, it was repeated a third time. (3) Blinding is not possible in this kind of intervention because it is not feasible to hide the presence of 4 MIs or 2 SIs in a participant’s mouth from either the participants or the assessor. However, the participant’s awareness that he had MIs instead of SIs does not affect resorption measurements. (4) The unevenness in group distribution and the small sample size can be attributed to the COVID pandemic, high costs, and the difficult import regulations of our country. Nonetheless, a clinical trial conducted by Taylor and Francis suggested that this uneven distribution has a minor impact on the interpretation of statistical analysis results [55]. While the sample size was determined using the G-Power _3.1_ program with an 80% power level, there is still a risk of a Type II error due to the limited sample size. Finally, as this is a short-term study (1.5 years), a longer follow-up would be necessary to evaluate long-term survival and the stability of marginal bone, and such extended studies should be considered in the future.

## Conclusion

1. The survival rate of 4 MIs (3 mm or 2.5 mm diameter) and 2 SIs (3.75 mm diameter) retained mandibular overdentures is 100% after 1.5 years.

2. The success rate after 1.5 years is 90.91–90.63-90% in the S, M3, M2.5 groups, respectively.

3. Mean MBLC did not exceed 1.5 mm in all groups after 1.5 years. However, in general, the mean MBLC increased and continued over time with a decrease in the implant’s diameter. The larger the diameter, the better the radiographic results.

4. Mandibular overdentures retained by 4 MIs are a successful treatment for edentulous patients. However, mandibular overdentures retained by 2 SIs have shown better radiographic results and success rate.

## Supplementary information

CONSORT-2010-Checklist

## Data Availability

The datasets used and analyzed during the current study are available from the corresponding author on reasonable request.
